# Linkage between Neighborhood Social Cohesion and BMI of South Asians in the Masala Study

**DOI:** 10.1155/2020/7937530

**Published:** 2020-01-07

**Authors:** Gagandeep Gill, Nicola Lancki, Manjit Randhawa, Semran K. Mann, Adam Arechiga, Robin D. Smith, Samuel Soret, Alka M. Kanaya, Namratha Kandula

**Affiliations:** ^1^School of Public Health, Loma Linda University, Loma Linda, CA, USA; ^2^Northwestern University Feinberg School of Medicine, Departments of Medicine and Preventive Medicine, Chicago, IL, USA; ^3^National Environmental Health Association, Denver, CO, USA; ^4^School of Behavioral Health, Loma Linda University, Loma Linda, CA, USA; ^5^University of California, San Francisco, CA, USA

## Abstract

**Introduction:**

South Asians in the United States have a high prevalence of obesity and an elevated risk for cardiometabolic diseases. Yet, little is known about how aspects of neighborhood environment influence cardiometabolic risk factors such as body mass index (BMI) in this rapidly growing population. We aimed to investigate the association between perceived neighborhood social cohesion and BMI among South Asians.

**Methods:**

We utilized cross-sectional data from the MASALA study, a prospective community-based cohort of 906 South Asian men and women from the San Francisco Bay area and the greater Chicago area. Multivariable linear regression models, stratified by sex, were used to examine the association between perceived level of neighborhood social cohesion and individual BMI after adjusting for sociodemographics.

**Results:**

Participants were 54% male, with an average age of 55 years, 88% had at least a bachelor's degree, and the average BMI was 26.0 kg/m^2^. South Asian women living in neighborhoods with the lowest social cohesion had a significantly higher BMI than women living in neighborhoods with the highest cohesion (*β* coefficient = 1.48, 95% CI 0.46–2.51, *p*=0.02); however, the association was not statistically significant after adjusting for sociodemographic factors (*β* coefficient = 1.06, 95% CI −0.01–2.13, *p*=0.05). There was no association between level of neighborhood social cohesion and BMI in South Asian men.

**Conclusion:**

Perceived neighborhood social cohesion was not significantly associated with BMI among South Asians in our study sample. Further research is recommended to explore whether other neighborhood characteristics may be associated with BMI and other health outcomes in South Asians and the mechanisms through which neighborhood may influence health.

## 1. Introduction

Obesity rates continue to rise in the United States (US), with some studies estimating that more than 78 million adults, or 1 in 3 adults, in the United States were obese in 2009-2010 [1, 2]. Obesity is linked to multiple preventable diseases and some of the leading causes of preventable deaths including type 2 diabetes, heart disease, stroke, and certain types of cancer [1, 3]. Cardiovascular disease accounts for nearly a quarter (23%) of the deaths of the South Asian and is the leading cause of death in populations aged 45 and above [4]. South Asians' incidence rate is 6 per 1,000 population and 11.2% population 12.2%, and 10.0% for type 2 diabetes [4]. The staggering rates of obesity across the US are a significant public health concern with significant biomedical, psychosocial, and economic implications [1]. While obesity is a serious problem for many people in general, it does affect some groups more than others [1].

Together, South Asians (from India, Pakistan, Bangladesh, Nepal, and Sri Lanka) make up one of the largest and fastest growing immigrant Asian subgroups in the United States [3]. While Asian Americans overall have a relatively low body mass index (BMI) compared with other race/ethnic groups in the US, South Asians specifically have the highest rates of overweight/obesity among all Asians in the US [5, 6]. In addition, South Asians are more prone to develop abdominal fat, [7, 8] have lower lean body mass, and have a higher proportion of overall body fat than non-Hispanic whites [7, 9]. Accordingly, South Asians have high rates of obesity-related chronic disease, such as type 2 diabetes and cardiovascular disease [8, 10, 11]. Since South Asians are a rapidly growing population in the US, it is important to identify the social and cultural factors that may underpin the increased obesity and related risks.

Given the continuing population trends of rising obesity rates and the serious health implications, there has been a growing focus on better understanding the related social determinants of health, and specifically, on how neighborhood environment influences individual's overall well-being and weight fluctuation [12]. While the association between neighborhood-built environment and obesity risk has been widely examined, studies suggest neighborhood social context may be equally important in understanding obesity risk [12, 13]. One important feature of the neighborhood social environment is social cohesion, which captures a central aspect of a neighborhood's social environment. In previous studies, neighborhood social cohesion has been defined by constructs such as perceived connectedness, solidarity, and shared resources that allow people to act together [13]. Studies in other racial/ethnic groups suggest that social cohesion may mediate the effects of other neighborhood factors [14], such as residential segregation and poverty, on obesity. In addition, there may be differential effects in how social cohesion impacts obesity risk in different racial and ethnic groups [15]. Higher neighborhood social cohesion was associated with lower obesity risk in non-Hispanic whites [14], but the literature on neighborhood cohesion and obesity risk in racial/ethnic minorities is sparse and inconsistent [16]. A study of South Asian and white European men in the United Kingdom found that South Asian men lived in more disadvantaged neighborhoods with lower social cohesion, and they also had a higher waist circumference than white men [17]. Even less is known about neighborhood social cohesion and women's health. In a prior paper, neighborhood social cohesion was associated with hypertension prevalence in South Asian women in the U.S., but not men, underscoring the need to examine the applicability of social cohesion in the context of sex [18].

The existing literature has highlighted the need for research that focuses on the influence of the neighborhood on South Asian men and women' health and more specifically on how social cohesion may link to obesity and obesity-related factors [19]. In response to this need, using data collected as part of the community-based Mediators of Atherosclerosis in South Asians Living in America (MASALA) study, we examined the association between perceived neighborhood social cohesion and individual BMI. We hypothesized that higher levels of neighborhood social cohesion would be associated with a lower BMI.

## 2. Methods

### 2.1. Participants

The MASALA study is a prospective community-based cohort of South Asian men and women from two clinical sites (San Francisco Bay area at the University of California, San Francisco and the greater Chicago area at Northwestern University). The baseline MASALA study examination was conducted by trained bilingual (English, Hindi, or Urdu) study staff from October 2010 through March 2013, and a total of 906 participants were enrolled [20]. For participants to be eligible for MASALA, participants had to be of South Asian ancestry and have at least three grandparents born in one of the following countries: India, Pakistan, Bangladesh, Nepal, or Sri Lanka, be between the ages of 40–84 years, and be able to speak and/or read English, Hindi, or Urdu [20–23] Exclusion criteria included a physician-diagnosed heart attack, stroke or transient ischemic attack, heart failure, angina, use of nitroglycerin, a history of cardiovascular procedures, current atrial fibrillation, active treatment for cancer, life expectancy < 5 years due to a serious medical illness, impaired cognitive ability, plans to move out of the study region in the next 5 years, and living in a nursing home. The study protocol was approved by the institutional review boards at the University of California, San Francisco, Northwestern University, and Loma Linda University.

### 2.2. Measures

#### 2.2.1. Predictor Variable

Our primary independent variable of interest was perceived neighborhood social cohesion, which was measured using a five-item Likert scale tool ranging from 1 (strongly agree) to 5 (strongly disagree) that had been previously validated [23–25], Respondents were asked to report their levels of agreement with the following statements: (Item 1) “people around here are willing to help their neighbors,” (Item 2) “people in this neighborhood generally do not get along with each other,” (Item 3) “people in this neighborhood can be trusted,” (Item 4) “people in this neighborhood do not share the same values,” and (Item 5) “most people in this neighborhood know each other” [18, 21, 23]. Items 1, 3, and 5 were “positive” constructs; therefore, we had to reverse code so that a higher score would indicate higher level of social cohesion. Items 2 and 4 are worded as “negative” constructs, so the strongly agree score of 1 does indicate lower levels of social cohesion. Cronbach's alpha for the items was 0.65.

We used principal component factor analysis with orthogonal rotation on the five items of the neighborhood questionnaire to construct the measure of social cohesion [16]. The first factor had an eigenvalue >1 and explained 43% of the variance. The results of the principal component factor analysis, factor loadings for factor 1, and scoring coefficients are shown in Supplementary [Supplementary-material supplementary-material-1]. The factor score was created as the standardized weighted average using the scoring coefficients from Supplementary [Supplementary-material supplementary-material-1] and have a mean 0 and standard deviation 1. Factor scores were then divided into tertiles and defined as lowest (first tertile), middle (second tertile), and highest (third tertile) levels of neighborhood social cohesion.

#### 2.2.2. Outcome Variable

The primary outcome was BMI, which was calculated from measured weight in kilogram divided by height in square-meter. Participant weight was measured on a standard balance beam scale or digital weighing scale, and height was measured using a stadiometer. We used BMI as a continuous variable to examine the association of BMI with social cohesion tertiles.

#### 2.2.3. Covariates

Our sociodemographic covariates included age, education, income, and marital status. Age was kept as a continuous variable in the model to preserve accuracy. Highest educational attainment was categorized as [1] less than a Bachelor's degree, [2] Bachelor's degree, and [4] more than a Bachelor's degree. In the original dataset, household income had 15 categories which we collapsed into three categories: [1] less than $50,000, [2] $50,000-$99,999, and [4] $100,000 or more. Marital status was also dichotomized as [1] married or living as married or [2] others (single, divorced, separated, or widowed).

### 2.3. Statistical Analysis

We first used descriptive statistics to assess sociodemographic characteristics (age, BMI, income, education, and marriage) by neighborhood social cohesion tertiles stratified by sex. Theoretical and empirical evidence suggests that women are more likely to be influenced by community social environment [24–27]; therefore, we conducted all analyses stratified by sex. Mean ± standard deviation or frequencies and percentages were presented. We compared sociodemographic characteristics by neighborhood social cohesion tertile using Chi-squared tests for categorical variables and ANOVA for continuous variables. Linear regression models stratified by sex were created to examine the associations between the categorical social cohesion tertile (reference category was high neighborhood cohesion) and continuous BMI outcome. For this analysis, we built two different models: model 1, where we examined the relationship between neighborhood cohesion tertile and BMI without adjusting for any covariates and model 2, where we adjusted for covariates (age, education, family income, and marital status). Statistical analyses were performed using SPSS Version 22 [28], and a two-sided *p* value of <0.05 was considered to be statistically significant.

## 3. Results

Characteristics of MASALA study participants by neighborhood cohesion tertile stratified by sex are shown in Table 1. There were differences in average BMI across neighborhood social cohesion tertile (BMI_lowest social cohesion tertile_ = 27.3 kg/m^2^, BMI_middle social cohesion tertile_ = 26.4 kg/m^2^, and BMI_highest social cohesion tertile_ = 25.9 kg/m^2^, *p*=0.015). This was not evident in men (BMI_lowest social cohesion tertile_ = 26.3 kg/m^2^, BMI_middle social cohesion tertile_ = 26.1 kg/m^2^, and BMI_highest social cohesion tertile_ = 26.6 kg/m^2^, *p*=0.480). Among both men and women, there were differences in the distribution of education category, income category, and marital status by neighborhood social cohesion tertile (see Table 1).

In unstratified analyses, neighborhood social cohesion tertile was not associated with BMI in South Asian adults (*β*_lowest vs highest_ = 0.58, 95% CI (−0.11, 1.26), *p*=0.099; *β*_middle vs highest_ = -0.02, 95% CI (−0.68, 0.65), *p*=0.962) from the unadjusted model.

In models stratified by sex, in the unadjusted models, living in neighborhoods with the lowest social cohesion tertile compared with the highest social cohesion tertile was associated with a higher BMI in women (*β* = 1.48, 95% CI (0.46, 2.51), *p*=0.02), but not in men (*β* = −0.31, 95% CI (−1.22, 0.61), *p*=0.51) (refer Table 2). For women, this association was no longer statistically significant after adjusting for age, education, income, and marital status (*β* = 1.06, 95% CI (−0.02, 2.13), *p*=0.05) (refer Table 2). In the adjusted model in men, education and age were associated with BMI, having less than a bachelor's degree compared with having more than a bachelor's degree being associated with higher BMI (*β* = 1.67, 95% CI (0.29–3.05), *p*=0.020) and increasing age associated with lower BMI (*β* = −0.06 for each year increase in age, 95% CI (−0.10 to −0.03), *p*=0.001).

## 4. Discussion

The overall objectives of this study were to help fill a gap in existing literature and examine whether neighborhood social cohesion was associated with body mass index among South Asians in the US. Our study found that neighborhood social cohesion was not associated with BMI among South Asians in the MASALA study.

Studies looking at the association between neighborhood social cohesion and BMI have produced mixed results. A number of studies have found a significant association between higher neighborhood social cohesion and lower BMI [24, 25, 29]. In the MESA (Multi-Ethnic Study of Atherosclerosis) study, Mujahid et al. found that BMI was lower for women living in neighborhoods with a better social environment (which included social cohesion, aesthetics, crime, and safety), but that in men living in a neighborhood with a better social environment was associated with higher BMI [26, 29]. Literature from Carrillo et al. and others have had suggested that social capital and social cohesion can shape health behaviors and outcomes through “(i) informal control and the normalization of health‐related behaviors, (ii) collective efficacy, and (iii) exchange of social support” [30]. There remain knowledge gaps in how neighborhood environment and mediating pathways, such as social norms, may create or mitigate health risks in South Asians [31, 32].

Although we did find an association between neighborhood social cohesion and BMI in women, the association was attenuated after controlling for individual-level socioeconomic factors. There may be possible mediation; for example, higher education may be associated with a better diet, more exercise, and a lower BMI, but it may also be associated with living in a neighborhood with higher cohesion [33, 34]. Having a higher education and family income may help one to live in a place with higher social cohesion which influences factors on the pathway for BMI.

A number of other factors may have contributed to the null finding. First, it may be important to integrate social cohesion environment and other community-level factors. Our study population included residents in two major urban and outlying suburban areas in the US, where social cohesion with neighbors may be high, but the built environment (such as apartment buildings, or densely populated city dwellings/surroundings) may not allow for physical activity. Another important aspect of the neighborhood environment is neighborhood racial/ethnic composition which is an important correlate of obesity risk and is likely to impact social cohesion [32].

Additionally, the social cohesion measure itself could also have contributed to the null finding. Although our study uses a well-validated neighborhood social cohesion scale in general US population, the psychometric properties of this scale have not been evaluated in South Asian populations. The current study had a Cronbach's alpha of 0.65 for the social cohesion variable, which is acceptable in exploratory research, but should be further explored for improvement in future studies. To this end, additional questions regarding neighborhood social cohesion should be explored because South Asians may have different values and beliefs about what constitutes social cohesion compared with other racial/ethnic groups. In an analysis of National Health Interview Survey Data, the association of social cohesion with physical activity differed by race/ethnicity and was not associated with physical activity in several Asian American groups, including Asian Indians [35]. This suggests that the relevance of social cohesion on health must be examined within the context of race/ethnicity.

Furthermore, studies indicate that specific aspects of the neighborhood social environment, such as social capital, collective efficacy, and crime, are associated with obesity among both adults and children [25]. Safety, crime, and violence have been shown to have consistent associations with obesity; greater neighborhood safety is associated with lower BMI, and higher neighborhood crime is associated with higher BMI [32, 35, 36]. Specifically, neighborhood safety and crime might influence social cohesion among residents by limiting opportunities for social interaction and promoting distrust among residents [31, 32]. Studies have also identified physical activity as a potential pathway of the neighborhood safety and obesity association [33, 37, 38].

### 4.1. Limitations

Although the MASALA study cohort's demographics are similar to those of South Asians in the 2010 US Census, there are limitations [39]. The MASALA sample largely comprises Asian Indian immigrants living in the San Francisco and Chicago areas, and thus the results may not be generalizable to all South Asians in the U.S. Additionally, the study sample had a high SES, which does not capture the bimodal distribution of SES in different South Asian groups. Because this is a cross-sectional study, our ability to make causal inferences is limited. Longitudinal follow-up of the MASALA cohort will allow for further investigations about neighborhood effects and changes in BMI or other outcomes.

## 5. Conclusion

Our research provides important information regarding the relationship between social cohesion and obesity-related risk factors among South Asian Americans. We did not find an association between neighborhood social cohesion and BMI in South Asian adults. Future studies should examine other aspects of the built and social environment that may be associated with weight in South Asians and include individuals with greater variation in socioeconomic status and neighborhood environment.

## Figures and Tables

**Table 1:** Characteristics of the MASALA study participants by neighborhood social cohesion, 2010–2013, *N* = 906.

	Women (*N* = 420)					Men (*N* = 486)				
	Lowest tertile (*N* = 151)	Middle tertile (*N* = 149)	Highest tertile (*N* = 120)	Total	*p* value	Lowest tertile (*N* = 151)	Middle tertile (*N* = 207)	Highest tertile (*N* = 128)	Total	*p* value
	*N* (%)	*N* (%)	*N* (%)	*N* (%)		*N* (%)	*N* (%)	*N* (%)	*N* (%)	
Age, mean (SD)	55.58 (9.28)	54.53 (8.50)	52.63 (7.68)	54.37 (8.63)	0.0185	57.36 (9.47)	54.92 (9.90)	56.69 (10.35)	56.14 (9.93)	0.0555
BMI^*∗*^, mean (SD)	27.34 (4.19)	26.43 (4.35)	25.85 (4.02)	26.59 (4.30)	0.0156	26.29 (4.19)	26.08 (3.35)	26.60 (4.23)	26.28 (3.87)	0.4873
Income^*∗∗*^					0.0013					0.0133
Less than $50,000	38 (26.21)	20 (13.89)	12 (10.26)	70 (17.24)		39 (27.08)	28 (13.66)	18 (14.4)	85 (17.93)	
$50,000 to $99,999	33 (22.76)	29 (20.14)	19 (16.24)	81 (19.95)		27 (18.75)	39 (19.02)	22 (17.6)	88 (18.57)	
$100,000 or more	74 (51.03)	95 (65.97)	86 (73.50)	255 (62.81)		78 (54.17)	138 (67.32)	85 (68)	301 (63.5)	
Education					0.0036					
Less than a bachelor's degree	35 (23.18)	15 (10.07)	12 (10.0)	62 (14.76)		17 (11.26)	21 (10.14)	10 (7.81)	48 (9.88)	
Bachelor's degree	49 (32.45)	45 (30.20)	44 (36.67)	138 (32.86)		50 (33.11)	49 (23.67)	24 (18.75)	123 (25.31)	
More than a bachelor's degree	67 (44.37)	89 (59.73)	64 (53.33)	220 (52.38)		84 (55.63)	137 (66.18)	94 (73.44)	315 (64.81)	
Marriage					0.0234					0.0387
Married or living as married	120 (79.47)	133 (89.26)	107 (89.17)	360 (85.71)		141 (93.38)	202 (97.58)	126 (98.44)	469 (96.5)	
Others	31 (20.53)	16 (10.74)	13 (10.83)	60 (14.29)		10 (6.62)	5 (2.42)	2 (1.56)	17 (3.5)	

^*∗*^Missing for 3 participants (one woman and two men).

^*∗*^Missing for 26 participants (14 women and 12 men).

^*∗∗*^ *P* > 0.05.

**Table 2:** Association between neighborhood social cohesion and BMI stratified by sex, MASALA study (2010–2013).

Model 1 unadjusted items	Women (*N* = 419)				Men (*N* = 484)			
	Standardized coefficients *Β*	95% confidence interval for *β*		*p* value	Standardized coefficients *Β*	95% confidence interval for *β*		*p* value
		Lower bound	Upper bound			Lower bound	Upper bound	
BMI (constant)	25.85	25.09	26.62		26.60	25.93	27.27	
Neighborhood cohesion								
Lowest tertile	1.48	0.46	2.51	0.02^a^	−0.31	−1.22	0.61	0.51
Middle tertile	0.57	−0.46	1.60	0.27	−0.52	−1.38	0.33	0.23
Highest tertile	Ref	—	—	—	Ref	—	—	—

	Women (*N* = 405)				Men (*N* = 472)			
Model 2 adjusted items	Standardized coefficients *Β*	95% confidence interval for *β*		*p* value	Standardized coefficients *Β*	95% confidence interval for *β*		*p* value

BMI (constant)	27.77	24.69	30.84	<0.0001	29.98	27.57	32.39	<.0001
Neighborhood cohesion								
Lowest tertile	1.06	−0.01	2.13	0.05	−0.23	−1.16	0.70	0.63
Middle tertile	0.47	−0.58	1.52	0.38	−0.63	−1.49	0.22	0.15
Highest tertile	Ref	—	—	—	Ref	—	—	—
Age in years	−0.04	−0.09	0.01	0.15	−0.06	−0.10	−0.03	0.001^a^
Education								
Less than a bachelor's degree	1.25	−0.10	2.60	0.07	1.67	0.29	3.05	0.02^a^
Bachelor's degree	−0.16	−1.10	0.79	0.75	0.42	−0.44	1.28	0.34
More than a bachelor's degree	Ref	—	—	—	Ref	—	—	—
Income								
Less than $50,000	1.39	−0.01	2.78	0.05	−0.61	−1.82	0.60	0.32
$50,000 to $99,999	Ref	—	—	—	Ref	—	—	—
$ 100,000 or more	−0.10	−1.24	1.03	0.86	−0.14	−1.08	0.80	0.77
Marriage								
No (ref = yes)	0.35	−0.92	1.61	0.59	0.59	−1.42	2.61	0.56

^a^ *p* < 0.05.

## Data Availability

The Mediators of Atherosclerosis South Asians Living in America (MASALA) data used to support the findings of this study are restricted by the University of California, San Francisco, and Northwestern University in order to protect patient privacy. Data are available by request and approval by the MASALA steering committee for researchers who meet the criteria for access to confidential data and must require further addition IRB approval with a signed data use agreement. Please contact masala@ucsf.edu and masalastudy@northwestern.edu.

## Supplementary Materials

Supplementary Materials Supplementary Table 1: principal component factor analysis. Supplementary Table 2: factor loadings and unique variances. Supplementary Table 3: scoring coefficients (method = regression; based on varimax rotated factors).
