# Supplementary material for: Linkage between Neighborhood Social Cohesion and BMI of South Asians in the Masala Study
Source: J Obes. 2020 Jan 7;2020:7937530. doi: 10.1155/2020/7937530 (PMC6969985; doi:10.1155/2020/7937530)
Supplement: Supplementary Materials — Supplementary Table 1: principal component factor analysis. Supplementary Table 2: factor loadings and unique variances. Supplementary Table 3: scoring coefficients (method = regression; based on varimax rotated factors). [file 7937530.f1.pdf]

Supplementary Table 1. Principal component factor analysis

Factor	Eigenvalue	Difference	Proportion	Cumulative
Factor1	2.17081	1.17261	0.4342	0.4342
Factor2	0.9982	0.28263	0.1996	0.6338
Factor3	0.71557	0.07104	0.1431	0.7769
Factor4	0.64453	0.17363	0.1289	0.9058
Factor5	0.4709	.	0.0942	1

Supplementary Table 2. Factor loadings and unique variances

Item	Factor 1	Uniqueness
People around here are willing to help their neighbors	0.7955	0.3672
People in this neighborhood generally don't get along with each other	0.602	0.6376
People in this neighborhood can be trusted	0.6997	0.5104
People in this neighborhood do not share the same values	0.5719	0.673
Most people in the neighborhood know each other	0.5992	0.6409

Supplementary Table 3. Scoring coefficients (method=regression; based on varimax rotated factors)

Item	Factor1
People around here are willing to help their neighbors	0.36644
People in this neighborhood generally don't get along with each other	0.2773
People in this neighborhood can be trusted	0.32232
People in this neighborhood do not share the same values	0.26343
Most people in the neighborhood know each other	0.27603
